# Motor Assessment Timed Test (MATT): A New Timed Test to Assess Functional Mobility in Parkinson’s Disease Patients

**DOI:** 10.3390/jcm14020361

**Published:** 2025-01-09

**Authors:** Sergio Sebastia-Amat, Juan Tortosa-Martínez, Basilio Pueo

**Affiliations:** Health, Physical Activity and Sports Technology (HEALTH-TECH), Department of General and Specific Didactics, Faculty of Education, University of Alicante, 03690 Alicante, Spainbasilio@ua.es (B.P.)

**Keywords:** functional mobility, mobility impairment, neurodegenerative disorders, gait, balance, dual task, rehabilitation

## Abstract

**Background/Objectives**: Parkinson’s disease (PD) is a neurodegenerative disorder that significantly impairs motor function, leading to mobility challenges and an increased risk of falls. Current assessment tools often inadequately measure the complexities of motor impairments associated with PD, highlighting the need for a reliable tool. This study introduces the Motor Assessment Timed Test (MATT), designed to assess functional mobility in PD patients. **Methods**: A cross-sectional study was conducted involving 57 participants (38 men and 19 women) aged 44 to 84, diagnosed with idiopathic PD. Participants were recruited from three PD associations and underwent a series of assessments, including MATT, to evaluate gait, balance, and dual-task performance under conditions that reflect real-life challenges faced by individuals with PD. **Results**: MATT demonstrated excellent reliability with intra-rater reliability (ICC = 0.99), inter-rater reliability (ICC = 0.96–0.99), and test–retest reliability (ICC = 0.93–0.99). The coefficient of variation for total time and each segment ranged from 4.73% to 13.71%, indicating consistent performance across trials. The concurrent validity showed very high correlations with established tools such as the Timed Up and Go (TUG) test (ρ = 0.86, *p* < 0.001) and the Berg Balance Scale (BBS) (ρ = −0.83, *p* < 0.001), among others. Only 7.3% of participants reported difficulties in understanding the MATT, predominantly those in advanced stages of the disease. In addition, 23.6% of participants experienced significant challenges in performing the test, particularly individuals with lower Mini-Mental State Examination (MMSE) scores (≤ 26) and more advanced disease progression. **Conclusions**: MATT is a promising tool for assessing motor complications in PD, offering a comprehensive evaluation of functional mobility. Its implementation in clinical practice could enhance the management of PD, facilitating tailored interventions and improving patient outcomes.

## 1. Introduction

Parkinson’s Disease (PD) involves a wide range of motor anomalies, such as disturbance in gait, balance, and posture [1,2]. Among these, gait disturbances are particularly prevalent and disabling, significantly affecting the quality of life of individuals with PD [3].

Gait disturbances can be divided into two types: continuous and episodic [4]. Continuous gait disturbances refer to persistent alterations in the walking pattern that seem, at first glance, relatively stable from one step to the next, whereas episodic gait disturbances occur sporadically and intermittently, appearing in an unpredictable and unexplained manner [5]. Episodic gait disturbances include phenomena such as festination, start hesitation, and Freezing of Gait (FOG). FOG, in particular, affects approximately 50% of people with PD, with its prevalence increasing as the disease progresses [6]. FOG is defined as “a brief, episodic absence or marked reduction of forward progression of the feet despite the intention to walk” [7] and is often described by patients as the sensation of their feet being “glued to the floor” [8]. FOG and other motor complications commonly manifest during specific activities, such as initiating walking, reaching a target, turning (e.g., 180° or 360°), navigating obstacles, crossing narrow spaces, or performing dual tasks [9,10]. These difficulties are further influenced by external and internal factors, including visual inputs, time constraints, attention, anxiety, and stress, all of which can exacerbate motor impairments [11]. In addition to gait disturbances, balance impairments represent a major issue for individuals with PD, affecting both older patients and younger individuals in the advanced stages of the disease [12]. The high prevalence of balance disturbances in the PD population significantly increases the risk of falls and can be disabling, even for individuals in the early stages of the disease [13].

These axial features are key for functional mobility, understood as the ability to perform functional tasks and engage in activities of daily living [14], and they are typically responsible for the increase in the risk of falls and dependence in this population [15,16]. Thus, functional mobility assessment is relevant for the diagnosis and prognosis of PD, providing valuable insight into the disease evolution, treatment effects, and motor performance of these patients [17]. Due to these considerations, timely and accurate identification of PD-related symptoms is essential for reducing risks associated with mobility impairments and enhancing quality of life [18].

Two types of measurements are currently in use for the evaluation of functional mobility in PD, subjective and objective [17]. Subjective methods, such as scales or questionnaires, rely on self-assessments by patients, rater-based interviews, or visual assessments. Despite their strong clinimetric properties, these tools rely on the subjective assessments of patients or health professionals to evaluate heterogeneous symptoms. This reliance can introduce potential errors when converting these assessments into numerical values [19].

To improve the accuracy of the diagnosis, most objective methods use technology devices that offer quantifiable measurements. However, the clinical application of these instruments is often limited due to the time required for testing and data processing, as well as the high cost of the devices [20,21,22]. In this context, timed tests present a promising alternative. These tests are simple and cost-effective tools that provide quantitative, objective scores for assessing PD patients [23]. Likewise, they are compatible with traditional rating scores and technology devices.

Timed tests are commonly used to identify bradykinesia, postural instability, and gait difficulties; detect episodes of FOG; evaluate functional mobility; predict fall risk; monitor disease progression; and assess the response to treatments or interventions [23,24,25]. While the most widely used timed tests have been validated in a PD population and exhibit good-to-excellent reliability, they still show limitations in assessing the full range of PD-related motor characteristics [26].

The Timed Get Up and Go (TUG) test is one of the most frequently used tools for assessing functional mobility in PD patients and is the only timed test classified as a recommended tool [27]. However, it is important to note that the TUG test was not originally designed for patients with PD and its complex motor characteristics, which raises questions about its suitability for this population and makes certain limitations difficult to address. Various attempts have been made to address the limitations of the TUG test, not only for populations with PD [26] but also for other populations [28,29]. For example, the Expanded Timed Get Up and Go increases the total distance of the test and uses a multimemory stopwatch to assess subtasks [28], while the modified version of the Expanded TUG involves performing each of the subtasks in a series, with a pause and new instructions given before proceeding to the next subtask [29]. More recently, the L-test has been validated for use in PD [26], extending the total walking distance and incorporating turns in both directions compared to the TUG. However, the L-test also presents design challenges, such as asymmetry in the type and direction of turns, and it still lacks some of the more demanding tasks that are relevant for assessing motor impairments in individuals with PD. Additionally, the final score of the L-test may obscure difficulties encountered in specific subtasks.

Bloem et al. [18] reported that no instrument, whether subjective or objective, comprehensively and separately evaluates all relevant gait and balance characteristics in PD with both good clinical properties and adequate validity. Moreover, there is a lack of consensus regarding the most suitable instruments for monitoring functional mobility in PD patients. Therefore, they recommended the development of a specialized PD tool capable of evaluating the most significant motor disorders of the disease. In response to these needs, we aim to develop a timed test that is cost-effective, objective, easy to administer, valid, and reliable for assessing the components of functional mobility based on PD-related motor complications, both comprehensively and separately.

## 2. Materials and Methods

### 2.1. Design

A cross-sectional study design was used for this research.

### 2.2. Sample Size

Based on a pilot study, sample size calculation was performed using the G*Power 3.1.9.7 program. The criteria for sample size calculation were as follows: α = 0.05, 95% power, and a medium effect size (d = 0.5), considering a correlation test (two tails). The estimated sample size was at least 42 participants. Anticipating a 20% attrition rate, the target was to recruit a minimum of 51 participants.

### 2.3. Participants

A total of 88 patients, aged 44 to 84 years (56 men and 32 women) with confirmed idiopathic PD, were included in this cross-sectional study. Participants were recruited from three PD associations. Inclusion criteria were as follows: subjects diagnosed with idiopathic PD according to the clinical diagnostic criteria of the United Kingdom Parkinson’s Disease Society Brain Bank (diagnosed by a neurologist), above 18 years old, Hoehn and Yahr stage between 1–4, and self-reported antiparkinsonian drug treatment at a stable medication for at least 8 weeks prior to joining the study. Exclusion criteria were as follows: a history of traumatic brain injury or stroke, severe chronic obstructive pulmonary disease, a neurological disorder other than PD, myocardial infarction in the past 12 months, severe orthopaedic problems in the lower limbs, clinical diagnosis of dementia, or severe cognitive impairment (Minimental Status Examination Score, MMSE < 24) [30].

Sixty-one patients met the inclusion and exclusion criteria and were initially tested. Two participants were excluded due to the detection of comorbidities in the 8 weeks following the test session, and two others were excluded as they were unable to complete the evaluation session due to difficulties in remembering the sequence of the test, loss of balance during 360° static turns, or challenges overcoming obstacles. Consequently, the final sample size for analysis was 57 participants.

All testing procedures were conducted, while participants were in the “ON” state, 45–90 min after their first dopaminergic medication intake. All participants were able to ambulate independently, with or without a walking aid, and follow simple instructions. Participant characteristics and drug dosages are summarized in Table 1 (detailed in Supplemental Material S1).

Participants were fully informed about the aim of the study, benefits, and potential risks. Written informed consent was obtained after the project was approved by the research ethics committee of the University of Alicante (approval number: UA-2018-07-11, approved on 11 July 2018). The present study was conducted in accordance with the ethical standards of the Declaration of Helsinki (1975), as revised in 2013.

### 2.4. Measures

The different domains were evaluated using validated instruments. These instruments were chosen based on their widespread use and proven validity in PD research.

#### 2.4.1. Demographic Measures

Demographic characteristics collected included age, gender, BMI, education level, and disease duration. Education level was assessed by self-reporting the total years of formal education [31]. Disease duration was calculated from the date of the idiopathic PD diagnosis to the date of the physical exam in the study [25].

#### 2.4.2. Disease Severity

Disease severity was rated using the Hoehn and Yahr scale (H&Y) [32].

#### 2.4.3. Clinical Motor Assessment

A motor evaluation was conducted using the Spanish version of the modified Unified Parkinson Disease Rating Scale motor section (MDS-UPDRS III) [33].

#### 2.4.4. Fall History and Fear of Falling (FoF)

To determine fall history, participants, with the help of their relatives, were asked to recall the number of falls suffered within the previous 6 months. A fall was defined as “an unexpected event in which the participant comes to rest on the ground, floor, or lower level”. FoF was measured with the Spanish short version of the Falls Efficacy Scale International (short FES-I) [34]. The concept of FoF was defined as concerns, especially anxiety, to walk or mobilize.

#### 2.4.5. Cognitive Impairment

Cognitive function was evaluated using the Mini-Mental Status Examination Score (MMSE) [30]. This test was also used to ensure that participants understood the full information provided by researchers.

#### 2.4.6. Executive Function

The Trail Making Test (TMT) was used to assess executive function and working memory [35]. The TMT consists of two parts (TMT “A” and TMT “B”).

Participants were asked to connect numbers (Part A), or numbers and letters (Part B), as quickly as possible. The score for each part was the completion time, and the difference between parts (B-A) was also recorded. Part A evaluates attention and processing speed, while Part B assesses cognitive flexibility and sequential alternation.

#### 2.4.7. Physical Function and Motor Complications Assessment

Freezing of Gait Questionnaire (FOG-Q): a self-administered questionnaire that assesses the presence and severity of freezing of gait episodes in PD patients [36]. The questionnaire includes six items (range 0–24 points); higher scores indicate more severity.

Activities-specific Balance Confidence scale (ABC scale): a 16-item subjective questionnaire (range 0–100%) that rates the balance confidence of patients in daily living activities [37,38]. The score is obtained by adding the scores of individual items and then dividing by the total number of items. Higher scores mean more self-confidence.

Tinetti Scale “TS” (Total Score): a rating scale of 16 items (range 0–28 points) that assesses gait, balance performance, and fall risk [39,40,41]. It is composed of two subscales: Tinetti Gait Section “GS” (seven items, range 0–12 points) and Tinetti Balance Section “BS” (nine items, range 0–16 points). Higher scores indicate better performance.

Berg Balance Scale (BBS): a rating scale of 14 items (range 0–56 points) that assesses balance performance and fall risk [42,43,44]. Higher scores indicate better performance.

Functional Reach Test (FRT): a test designed to assess dynamic balance performance. The participant was asked to stand next to, but not touch, a wall and position the arm closest to the wall at 90 degrees of shoulder flexion with a closed fist. The assessor recorded the initial position at the level of the third metacarpal head on a yardstick. The participant was then instructed to reach as far forward as possible without moving from the ground. The location of the third metacarpal was recorded again. The test score was determined by calculating the difference between the initial (arm at 90° shoulder flexion) and final positions (maximum reach distance) [45]. A ruler was allocated on the wall to measure the difference in centimeters.

Posturographic analyses: posturographic measurements were collected using a baropodometric platform (FreeMed, Sensor Medica, Rome, Italy) and FreeStep software (FreeStep v.1.0.3, Sensor Medica, Rome, Italy). The FreeMed’s platform comprises an active surface of 400 × 400 mm and 8 mm thickness working at a sample frequency of 100 Hz. The following stabilometric parameters were used to express deviation of the center of pressure (CoP): total excursion (TE), defined as the total length of path covered by the COP, and mean speed (MS), defined as the average speed of COP over the course of the trial duration. The measurements were conducted in a barefoot standing condition, with the arms hanging next to the body and feet hip-width apart, in the preferred foot position [46,47]. Posturographic parameters were measured for 30 s under open (OE) and closed eye (CE) conditions [48].

Ten-meter walking test (10-MWT): Participants were asked to walk 10 m at maximum speed, without running [49]. The score of the test is the speed (m/s) obtained from the time needed to complete the distance.

Time Up and Go (TUG) test: Participants were asked to rise from a chair, walk 3 m, turn, walk back, and sit down [50].

Cognitive Time Up and Go (Cognitive TUG) test: Participants were required to count backwards aloud from 100 while performing the TUG test [51].

Three trials of each timed test were conducted, except for the TMT, which had only one trial. Three trials were also used to conduct posturographic analysis, both open and closed eyes conditions. The participants rested 1 min between trials and 5 min between tests. The average score of the three trials was used for data analysis.

#### 2.4.8. MATT (Motor Assessment Timed Test) Design

The MATT test was designed in three different segments to measure the most important motor domains related to functional mobility, gait and balance, including specific challenging tasks for PD patients [14,52,53,54]. Gait and balance problems are exacerbated when a concurrent task is introduced [55]. The ability to perform dual tasks is impaired in PD due to deficits in cognitive function and the neurological systems responsible for motor control, which has a detrimental impact on functional mobility and quality of life [56,57]. Dual tasks can vary in type, domain, and difficulty. Several studies have suggested that cognitive tasks have a significant impact on walking performance in PD patients [58,59]. Therefore, we decided to include a cognitive dual task in Segment 3 of the MATT: walking and counting backward aloud starting from 100. The primary instruction was to perform both tasks simultaneously without prioritizing walking or counting.

The decision to include challenging tasks throughout the circuit was made to reflect the unpredictable and episodic nature of FOG. Furthermore, the inclusion of maximum speed conditions and the motor planning required to perform the circuit may provide valuable insights into motor complications.

In view of the previous considerations, the final structure of the MATT included segments for gait, balance, and dual-task performance:

- Segment 1: Gait and dynamic turn (360° in both directions).
- Segment 2: Balance control (two tandem steps and stepping over an obstacle) and static turns in a narrow space (360° in both directions).
- Segment 3: Dual task and walking through a narrow passage.

The materials required for the MATT test included a chair with armrests, six marker bars and four cones (to create a narrow space), three hoops for unipedal step and the 360° turn, as well as four small disc cones to indicate the turns, the start of the dual-task area, and the end of the circuit. Two cameras (Sony RX100 IV, Sony Corporation, Tokyo, Japan) were used to record the test. These cameras feature a high frame rate (960 fps), allowing for slow-motion recordings. The time to complete the MATT test was measured using a chronometer app with a lap counter (iPhone 7, iOS 12.4.8, Apple Inc., Cupertino, CA, USA), with timing accurate to the nearest 1/100 s.

### 2.5. Procedures

#### 2.5.1. Clinical Evaluation

Prior to data collection, eligible participants were invited to meet with an interviewer who conducted a structured interview to gather sociodemographic and clinical information. These data were used for initial screening. Subsequently, participants were evaluated by a neurologist, who administered the MDS-UPDRS (III) scale, which includes the H&Y scale. During the same week, two psychologists administered the MMSE. The information provided by the neurologist and psychologists was used for final screening.

#### 2.5.2. Questionnaire and Motor Scales Administration

Once participants were accepted into the study, various questionnaires were administered to collect information on daily living, fall history, and PD-related complications (Day 1). The TMT and motor scales were also administered within the same week (Day 2) under similar medication conditions (“ON” state, 45–90 min after the first dopaminergic medication intake). The questionnaires and motor scales were administered in the week prior to the administration of the motor timed tests.

#### 2.5.3. Motor Examination (Balance and Timed Test Administration)

Balance tests (posturography and FRT) and motor timed tests were performed on the same day (Day 3), with adequate recovery time between tests. Posturographic analysis was conducted first to avoid fatigue influence [60,61]. The FRT was then administered. Finally, the MATT test and validated timed tests were performed in the following order: MATT, 10 MWT, TUG, and Cognitive TUG.

#### 2.5.4. MATT Protocol

Initially, a researcher demonstrated and explained the test protocol. Participants then performed a practical trial to familiarize themselves with the protocol and to ask the rater any questions or address concerns. Due to the high risk of falling in PD patients, participants were instructed to perform the test as quickly as possible, but safely and without running, with the prompt: “Imagine you are going to be late for work”. For safety reasons, one researcher was close to the participant during the entire test but without providing additional guidance.

**Figure 1:**
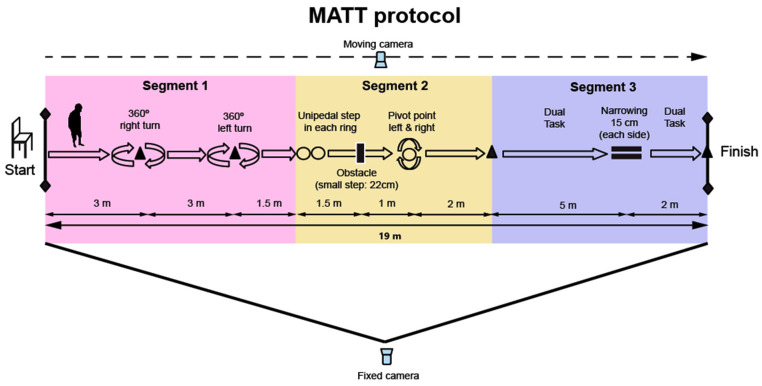
MATT test.

Protocol (step-by-step description):

1. Initial Position: Participants are instructed to sit in a chair with armrests at the starting point, as shown on the left side of Figure 1.
2. Start of the Test: The procedure begins when participants stand up from the chair following the “GO” command. The time is then recorded in seconds from this moment.
3. Walking and Right Turn: Participants walk 3 m to the first cone and perform a 360° right turn around the cone.
4. Left Turn: Participants continue walking straight and perform a 360° left turn upon reaching the second cone (3 m from the first).
5. Placing Feet on the Hoops: After passing the second cone, participants walk 1.5 m to the hoops, where they place their right foot on the first hoop and their left foot on the second hoop.
6. Overcoming the Obstacle: Participants then overcome an obstacle (22 cm high) without jumping.
7. Entering the Hoop and Performing Turns in a Restricted Space: After stepping over the obstacle, they enter a hoop 1 m ahead and perform two 360° turns inside the hoop (one clockwise and one counterclockwise).
8. Walking to the Next Cone: After exiting the hoop, participants walk 2 m to the next cone.
9. Counting Backwards and Crossing the Narrow Passage: Upon reaching the cone, participants begin counting aloud backwards from 100 while continuing to walk through a narrow passage (2 m long), marked by bars and cones, simulating a confined space (15 cm wide on each side of the body, at hip level).
10. End of the Protocol: Timing stops when participants tap the last cone, located 2 m from the midpoint of the narrow corridor.

All participants followed the same test protocol. The three segments were performed consecutively, without rest in between, as a single test. The rater provided verbal instructions when participants needed to count backwards (Segment 3). Three trials were conducted within the same test session, with 90 s of rest between them. Scores were not revealed to participants until after all tests were completed.

#### 2.5.5. MATT Assessment

A total of 171 trials were recorded using digital video with two synchronized cameras. The fixed camera was positioned parallel to the circuit, capturing the participants’ entire body, as shown in Figure 1. The moving camera, mounted on a three-axis gimbal stabilizer, was operated by a researcher who recorded the participants’ displacement in real time. In this case, the field of view was limited to the participants’ hips and lower extremities, except during the sit-to-stand task.

The MATT score was designed to be assessed without video documentation. However, due to the nature of the study, the two trained raters (Rater 1 and Rater 2) were unable to assess the participants in real time. Therefore, videos were used as a source of information to verify the reliability of the raters [62,63].

### 2.6. Clinimetric Properties

#### 2.6.1. Internal Consistency

Internal consistency was evaluated to determine the level of agreement or correlation between the three segments (Segment 1, Segment 2, and Segment 3), as their cumulative scores contribute to the overall MATT score. This type of reliability examines the extent to which each segment correlates with the others and adds meaningful information to the final score.

#### 2.6.2. Intra-Rater (Test–Retest) Reliability

Rater 1 evaluated the video recordings of each participant at two separate time points, with a 2-week interval between assessments. Videos were analyzed in the same sequence as they were performed. Test–retest reliability was calculated using the mean scores of the three trials (total time, Segment 1, Segment 2, and Segment 3), as well as paired scores for each trial and segment. Due to the study’s nature, test–retest reliability was assessed by Rater 1 using the same videos from the initial evaluation. Considering that the main source of the typical error arises from biological factors [64], such as changes in mental or physical state—common in the PD population—the use of the same videos offers a practical approach to specifically evaluate rater reliability.

#### 2.6.3. Inter-Rater Reliability

Two trained raters (Rater 1 and Rater 2) independently evaluated the video recordings offline in the same sequence as they were performed. To assess inter-rater reliability, the mean times for the three trials (total time, Segment 1, Segment 2, and Segment 3) were compared between raters. Additionally, individual trials and segments were analyzed to assess agreement for single measures. Both raters were blinded to each other’s ratings.

#### 2.6.4. Intra-Session Reliability

To evaluate intra-session reliability, each participant completed the MATT test three times on the same day. The total time and segment-specific scores were analyzed comprehensively across all trials (T1-T2-T3) and paired comparisons (T1-T2, T1-T3, and T2-T3). Intra-session reliability values were calculated separately for both Rater 1 and Rater 2.

#### 2.6.5. Development and Content Validity

The development and content validity of the MATT instrument were conducted following the guidelines proposed by Lynn [65] and Martinez–Martin et al. [66], adapted for this purpose. This process included two stages: “Development” and “Judgment and Quantification”.

The first stage of instrument development is identifying the target domains, selecting the sample task, and constructing the instrument. To select the target domains, an extensive literature review of the main tools used for the assessment of motor characteristics in PD patients was conducted. The second literature review was focused on the timed tests, analyzing their strengths and weaknesses. The literature review was also used to identify gaps in the design and application of timed tests in PD population. Hence, a first draft of the MATT instrument was developed based on the results of the previous literature reviews.

Stage 2 included two evaluations conducted by four judges/experts [65]. The experts were defined as professionals who work in practice or researchers who investigate in the PD field. Therefore, we decided to contact and include a neurologist specializing in motor disorders, two physiotherapists, and a sports science professional in the group of experts.

Previous to the first evaluation, the MATT tool was introduced to the group of experts, and the objectives were explained. Subsequently, the group of experts assessed independently the relevance, applicability, and suitability of the MATT. As a part of content validity, the experts were asked to suggest areas of improvement and identify areas of omission. The modifications proposed by each expert were collected and evaluated by the developer to modify the original test if deemed necessary.

A pilot study (*n* = 5) was conducted in order to detect and amend flaws in the MATT test. This included confirmation of target domain, assessment of participant and administrator burden, evaluation of patient understanding, and reduction of redundant tasks. This pilot study was finally used to obtain preliminary results of reliability and acceptability and also to calculate the sample size needed in the study.

After the pilot study, a second evaluation round was conducted with the same experts in order to re-evaluate the modified version of the test. A brief report of the modified version was also requested from each expert. Finally, the new version was resubmitted to obtain the final approval by the group of experts. The content validity process of the MATT instrument finished with the agreement of all members of the expert group.

#### 2.6.6. Content Validity

Concurrent validity was assessed by examining the association between the MATT test and other validated questionnaires and clinical tests targeting similar domains. Since no multi-domain timed test is considered a “gold standard”, we have utilized various validated tests in our analysis.

#### 2.6.7. Clinical Applicability

The time required to administer the MATT, from the start of the circuit explanation to the completion of the third trial, was recorded. Any falls or near-falls were also documented.

To assess participants’ understanding of the test, they were asked: “How complex did you find understanding the test”? To evaluate its difficulty, participants were asked: “How complex did you find performing the test”? Responses to both questions were rated on a Likert scale (1 = Very Easy, 2 = Easy, 3 = Medium, 4 = Difficult, 5 = Very Difficult).

### 2.7. Statistics

Descriptive statistics were presented as means and standard deviations. Data normality was assessed using the Kolmogorov–Smirnov test, and homogeneity of variance was tested with Levene’s test.

A one-way analysis of variance (ANOVA) was used to identify statistically significant differences between disease severity groups, with post-hoc comparisons conducted using the Bonferroni test. The significance level was set at *p* < 0.05.

Internal consistency among the segments of the MATT test was evaluated using Cronbach’s alpha for the mean time across Trials 1, 2, and 3. Additionally, Pearson’s correlation coefficient was used to analyze correlations between segments and the relationship of each segment with the total score. Intra-rater (rater 1) and inter-rater (raters 1 and 2) reliability were calculated using Cronbach’s alpha and the intraclass correlation coefficients (ICC) for Trials 1, 2, and 3 (ICC model 2,1: two-way random model) with absolute agreement and 95% confidence intervals. The familiarization trial was not analyzed. Cronbach’s alpha values ≥ 0.70 were considered acceptable [67]. ICC values were classified as follows: poor (<0.5), moderate (0.5–0.75), good (0.75–0.9), and excellent (>0.9) reliability [68].

The coefficient of variation (CV), expressed as a percentage (% CV), was calculated using the formula: CV (%) = 100·∑(SD_i/µ_i)_i_/*n*, where SD_i and µ_i are the standard deviations of the three repetitions of the ith subject, and *n* is the number of subjects. A CV <15% was considered acceptable [69].

Absolute reliability was assessed using the standard error of measurement (SEM) and the minimal detectable change score at a 95% confidence interval (MDC_95_). SEM was calculated as follows: SEM = SD × √ (1–ICC), where SD is the standard deviation of the measurements and ICC is the reliability coefficient. SEM%, independent of measurement units, was computed as: (SEM/$\bar{x}$) × 100, where $\bar{x}$ is the mean of the three trials The MDC_95_ is an estimation of a measure’s responsiveness, i.e., the ability of an outcome measure to detect “true” change, and it was obtained using the following equation: MDC_95_ = 1.96 × SEM × √2, where 1.96 is the z-score at the 95% confidence level and √2 is the correlation factor for repeated measurements. The MDC_95_% was determined by the following formulas: (MDC_95_/$\bar{x}$) × 100. An MDC_95_% < 30% was considered acceptable, and an MDC% < 10% was considered excellent [70]. Bland–Altman plots were also constructed for intra- and inter-rater reliability. The X-axis represented the mean of the scores, while the Y-axis showed the differences between scores [71]. 

Since intra-rater reliability was only assessed for rater 1, we decided to use the data of this rater for statistical calculations. Convergent validity was estimated using Pearson’s (r) and Spearman’s correlation coefficient (ρ), depending on data distribution. The magnitude of the correlation coefficients was interpreted following the augmented thresholds proposed by Hopkins et al. [72]: trivial (<0.1), small (0.1–0.3), moderate (0.3–0.5), high (0.5–0.7), very high (0.7–0.9), and nearly perfect (>0.9). A significance level of *p* < 0.05 was applied.

A repeated-measures ANOVA was conducted to evaluate whether scores differed across the three trials, potentially indicating learning or habituation effects. Pairwise differences were examined using Bonferroni-corrected paired *t-*tests.

Finally, the results of clinical applicability were expressed as percentage. A one-way ANOVA was performed to determine significant differences across disease severity groups, with Bonferroni post-hoc comparisons. The significance threshold was set at *p* < 0.05.

The Statistical Package for Social Sciences v. 26 (IBM Corp, Armonk, NY, USA) was used for data analyses. Bland–Altman plots were created with MedCalc statistical software (version 19.8, MedCalc Software, Mariakerke, Belgium). All data were recorded and encrypted in a password-protected spreadsheet software package.

## 3. Results

The mean time to complete the MATT was 55.11 s for the total sample. Detailed results, including mean time, standard deviation (SD), and significant differences, are presented in Table 2.

### 3.1. Reliability

#### 3.1.1. Internal Consistency

The internal consistency analysis revealed good agreement between the segments of the MATT test, with a Cronbach’s alpha of 0.846. Correlations between the segments and the total time of the MATT test are detailed in Table 3.

#### 3.1.2. Intra-Rater Reliability

Intra-rater reliability for Rater 1 was determined to be excellent for the total time and for all three segments of the MATT test (Cronbach’s alpha = 0.999, ICC = 0.998–0.999). Supplemental Material S2 provides comprehensive details about ICC values, 95% confidence intervals (CIs), coefficients of variation (CVs), standard error of measurement (SEM), and minimal detectable change (MDC_95_). For the total time of the MATT, the MDC_95_ was 3.37 s, indicating the smallest change in time that can be considered beyond measurement error. Bland–Altman plots (Supplemental Material S3) showed a high agreement between test–retest scores, with minimal systematic bias observed across all segments and the total time.

#### 3.1.3. Inter-Rater Reliability

Inter-rater reliability was also excellent for the total time and all three segments (Cronbach’s alpha = 0.995–0.999, ICC = 0.965–0.999). Additional information about inter-rater reliability is provided in Supplemental Material S4. The Bland Altman plots (Supplemental Material S5) showed excellent agreement between the three measurements of the MATT total time. The scattering plots were close to the mean line (equal to zero) and between the mean ± SD change (95% confidence interval). Although some outliers were detected, the reliability values remained excellent.

#### 3.1.4. Intra-Session Reliability

For Rater 1, ICC values were excellent for the total time (0.981–0.989) and for all three segments (0.931–0.991). CV values for total time and segments indicated low variability, ranging from 4.42% to 13.42%. Similar results were observed for Rater 2, with ICC values ranging from 0.980 to 0.988 for the total time, and 0.931 to 0.990 for the three segments. CV values for Rater 2 ranged from 4.73% to 13.71%. Detailed data on SEM, SEM%, MDC_95_, and MDC_95_% are available in Supplemental Material S6.

Bland–Altman plots (Supplemental Material S7) showed the distribution of the differences between the three trials of the MATT test (total time) conducted within the same day for Rater 1. A high level of agreement was found since the majority of paired measurements fell inside the 95% limits of agreement, depicted in dashed lines.

#### 3.1.5. Concurrent Validity

##### Correlation Analysis

The concurrent validity was investigated by correlating the total time and the different segments of the MATT with the different validated tests for each domain. Detailed correlation coefficients and significance values are available in Supplemental Material S8.

- Total Time: Very high correlations were found with the TUG (ρ = 0.86, *p* < 0.001) and BBS (ρ = −0.83, *p* < 0.001). Additionally, high correlations were found with the disease stage “H&Y scale” (ρ = 0.66, *p* < 0.001), the motor examination “MDS-UPDRS III” (ρ = 0.60, *p* < 0.001), the history of falls “6-M retrospective falls” (ρ = 0.65, *p* < 0.001), and the questionnaire of freezing of gait “FOG-Q” (ρ = 0.50, *p* < 0.001).
- Segment 1: Very high correlations were found with gait speed (10-MWT: r = −0.88, *p* < 0.001) and also with functional mobility (TUG: ρ = 0.89, *p* < 0.001). A high correlation was found between Segment 1 and the Tinetti Gait Section (ρ = −0.64, *p* < 0.001).
- Segment 2: Very high correlation was found with the specific balance scale (BBS: ρ = −0.84, *p* < 0.001). Moreover, high correlations were found with the Tinneti Balance section (ρ = −0.68, *p* < 0.001), the Tinneti total score (ρ = −0.74, *p* < 0.001), and the ABC scale: ρ = −0.69, *p* < 0.001). Nevertheless, a moderate correlation was found between Segment 2 and the FRT (r = −0.40, *p* < 0.001) and between this segment and the stabilometry variables, both open (TE COP: ρ = 0.33, *p* = 0.015; MS COP: ρ = 0.34, *p* = 0.011) and closed eye conditions (TE COP: ρ = 0.41, *p* = 0.002; MS COP: ρ = 0.41, *p* = 0.002).
- Segment 3: Very high correlation was found with the Cognitive TUG (ρ = 0.84, *p* < 0.001). Moreover, Segment 3 showed high correlations with the executive function (TMT “A”: ρ = 0.53, *p* < 0.001; TMT “B”: ρ = 0.58; *p* < 0.001; TMT “B-A”: ρ = 0.55; *p* < 0.001).

#### 3.1.6. Learning Effect

A one-way repeated-measures ANOVA detected significant differences between Trials 1 and 2 (*p* < 0.001) and Trials 1 and 3 (*p* < 0.001). No significant differences were found between Trials 2 and 3 (*p* = 0.999), suggesting a learning effect occurred primarily between Trials 1 and 2. The third trial provided minimal additional information.

#### 3.1.7. Clinical Applicability

MATT can be set up in approximately 5 min. The mean time to administer a trial was approximately 1 min, and 8 min are needed to explain the MATT test, conduct the practical trial, and record Trial 1. Finally, approximately 12 min were needed to administer the three trials. Depending on the score obtained in the Hoehn and Yahr Scale, it will take up to 15 min in the later stage disease (stage IV).

**Figure 2:**
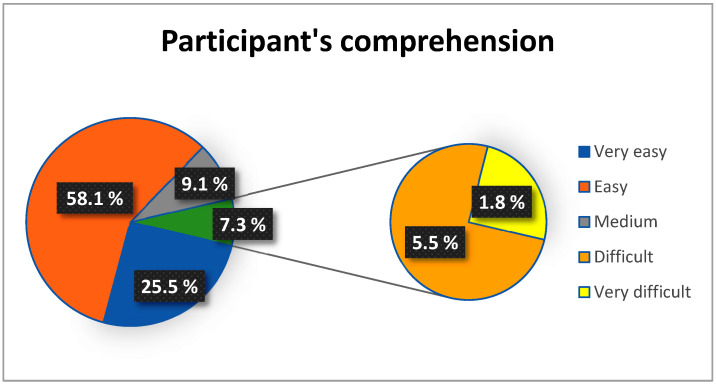
Percentage distribution of participants according to level of comprehension.

**Figure 3:**
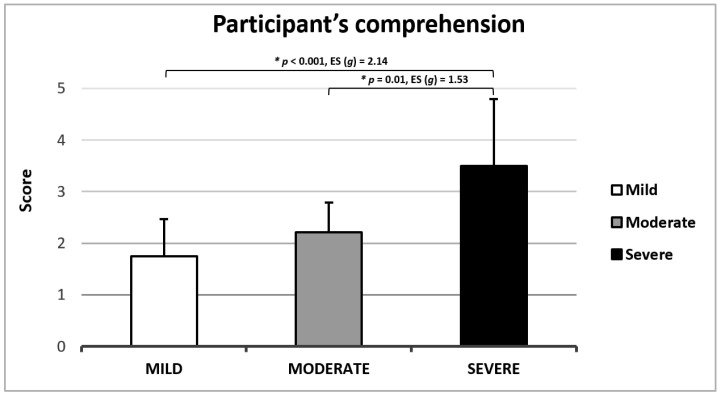
Participant’s comprehension scores according to disease stage.

Only 7.3% of participants reported high difficulty in understanding the MATT test (Figure 2). The participants that reported these difficulties were participants with the lower MMSE (score = 24) and higher disease stage (Figure 3).

**Figure 4:**
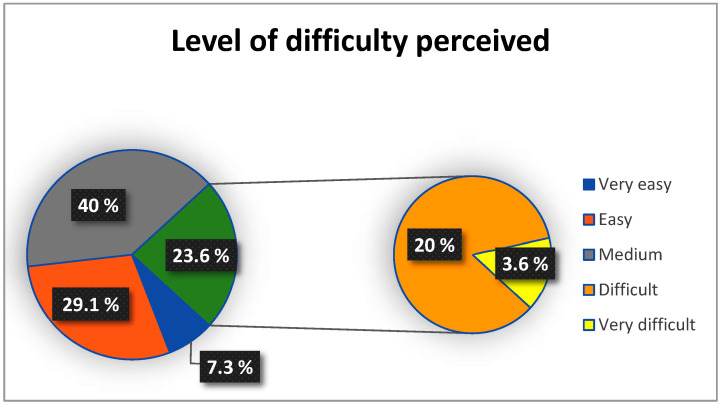
Percentage distribution of participants according to difficulty perceived.

**Figure 5:**
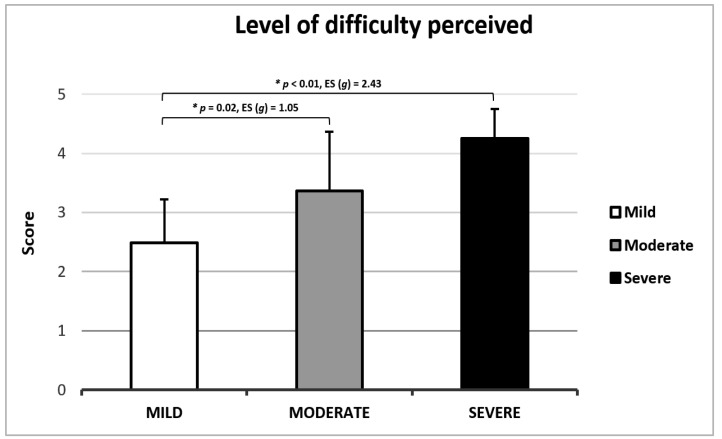
Level of difficulty perceived according to disease stage.

Regarding the perceived difficulty in performing the MATT test, 23.6% of participants reported a high level of difficulty (Figure 4). Participants who experienced greater challenges in completing the test were characterized by lower scores on the MMSE (≤ 26) and a more advanced disease stage (Figure 5).

Finally, a comprehensive summary of the clinimetric properties is presented in Supplemental Material S9. This document provides an overview of the properties evaluated and analyzed, as well as those that warrant further investigation.

## 4. Discussion

The primary aim of this study was to develop and validate a novel objective tool, the MATT test, to assess functional mobility and motor complications in PD. MATT was designed as a comprehensive and domain-specific evaluation of these features, including, in the test, the most challenging motor tasks for PD patients, and trying to overcome the limitations of previous tests.

The main finding of this research was that the MATT test is a valid and reliable instrument for measuring functional mobility and identifying difficulties in performing activities of daily living. Notably, the test’s segmented structure enabled the separate evaluation of gait, balance, and dual-task performance. A unique advantage of the MATT test is its ability to assess various motor domains within a single test, distinguishing it from existing tools.

The MATT test displayed good internal consistency, with each segment contributing significant information to the total time score, ensuring the comprehensive evaluation of functional mobility. The clinimetric analysis revealed excellent intra-rater reliability for the total score and individual segments. Importantly, a change in the MATT time exceeding 3.37 s was identified as a true performance change rather than a measurement error.

Inter-rater reliability was also excellent, with ICC values ranging from 0.965 to 0.999 among experienced raters. The fact to design a simple timed test with clear instructions and visual references that help raters to accurately assess patients, along with the experience of raters and the use of videos for the assessment process, could explain the high inter-rater reliability found.

The high agreement between trials within the same session (ICC > 0.931) and the acceptable values for the CV (< 15%) showed a very good intra-session reliability. Interestingly, Segment 3 showed the highest CV values. This may be due to dual-task interference or the fatigue effect. Considering the high intra- and inter-rater reliability mentioned above, the results of the within-session reliability can also be interpreted as very good within subject variability.

### 4.1. Functional Mobility

The MATT test demonstrated strong concurrent validity, evidenced by its high correlation with the TUG test (ρ = 0.86, *p* < 0.001), the most frequent tool used to assess functional mobility in PD populations [52]. In the same way, a very high correlation was found between the MATT test and the BSS (ρ = −0.83, *p* < 0.001). As expected, the mean completion time for the MATT test was lower in participants with mild PD (40.48 ± 11.68 s) compared to those with moderate (74.57 ± 53.45 s) or severe stages (122.32 ± 47.67 s). Consistent with these findings, previous studies have reported differences in motor performance using validated timed tests [73], as functional mobility in the PD population progressively declines with disease advancement [14]. These results suggest that the MATT test is capable of assessing functional mobility in PD patients. In contrast to the TUG test, the MATT test allowed the rater to assess, compressively and separately, the functional mobility under the most challenging conditions for this population. Moreover, the requirement of motor planning in consecutive tasks could make visible some motor problems such as FOG, which may be masked during the TUG performance as it is a very short and straightforward test.

### 4.2. Gait and Dynamic Balance

Segment 1 of the MATT test, which was designed to assess gait performance and dynamic turning, showed high to very high correlations with gait speed (10-MWT) and the functional mobility test (TUG). On the one hand, the very high correlation observed between Segment 1 and the TUG test may be attributed to the similar structure and distance in both tests. Despite these similarities, Segment 1 incorporated two 360° turns in both directions (clockwise and anticlockwise), which are particularly challenging for PD patients [9,74], whereas the TUG test only assessed a 180° turn in a preferred direction, potentially masking impairments, especially in patients with unilateral affectation. On the other hand, the L test, proposed as a more appropriate tool for assessing PD patients’ mobility, still presents controversial issues, such as the asymmetry in turn type and direction. These limitations in the L-test design appear to have been addressed in Segment 1.

Segment 2, designed to assess dynamic balance performance, demonstrated high-to-very high correlations with gold-standard tools, such as the BBS and the Tinetti Balance Scale (both total score and balance section score). The inclusion of 360° turns in narrow spaces in Segment 2 is particularly challenging for patients with PD, as this task demands a high level of balance control and coordination. Compared to 90° or 180° turns used in other tests, 360° turns present a more complex challenge, further aiding in the detection of balance impairments and providing a more accurate assessment of functional mobility in this population [75,76].

Additionally, a high correlation was found with the ABC scale, which measures balance confidence in performing various activities of daily living without losing balance. A key advantage of Segment 2 is that it is based on an objective parameter, unlike clinical assessment scales that rely on the rater’s or patient’s subjective information. The patient’s ability to recall or rate events, as well as the expectations or prior experiences of the rater, can influence the scores on these scales or questionnaires [77]. Furthermore, it has been observed that subjective clinical scales may have limitations in detecting turning anomalies, an issue that does not appear to be present with timed tests, especially when they are instrumented [78]. This same study indicated that the BBS was the scale with the highest detection capacity, which coincidentally is also the scale with the strongest correlation with Segment 2 of the MATT test in our study.

When comparing the results of Segment 2 with objective clinical balance tests, moderate correlations were observed with the FRT and static posturography variables. These lower correlations may be attributed to the fact that Segment 2 of the MATT test evaluates balance under dynamic conditions, while the FRT and static posturography tests assess balance under static conditions. The literature suggests that dynamic balance assessments are more appropriate for evaluating balance and fall risk in PD populations [31,79].

Segment 3, designed to assess walking ability under cognitive dual-task conditions, showed a high correlation with the Cognitive TUG test, indicating that both tests largely assess the same phenomenon. Similarly, Segment 3 showed a high correlation with the MMSE, as dual-task performance can be influenced by cognitive status [80,81]. High correlations were also found between Segment 3 and executive function performance (TMT variables). Previous research has shown that deficits in executive function are linked to gait impairment and episodes of FOG [82,83].

Regarding clinical applicability, the MATT test required a relatively short administration time (approximately 8 min), which is shorter than BBS (10–20 min), the Tinetti Balance Scale (5–10 min), and the ABC scale (10–20 min); similar to the 6-Minute Walk Test (<10 min); and slightly longer than the 10-MWT and TUG (5–10 min) [18,52,84]. Therefore, the primary advantage of the MATT test is its ability to assess multiple motor domains in a single test, thus significantly reducing the total time required for a comprehensive motor function assessment.

Repeated ANOVA and post-hoc Bonferroni tests revealed a learning effect between Trials 1 and 2 following a practice trial. These findings suggest that two timed trials are necessary to ensure performance stability for the MATT test in a PD population. Similar results have been reported in the literature, demonstrating that multiple trials improve the accuracy of the TUG test in various populations [63,85].

Concerning the participants’ perception while taking the test, both the comprehension level and the difficulty of performing the test were deemed adequate. However, a small percentage of participants reported difficulties understanding or performing the MATT test, primarily those with advanced stages of the disease and cognitive status near the eligibility threshold. Therefore, we recommend using the MATT test for mild-to-moderate PD patients, with further investigation needed for patients in more severe stages of the disease.

## 5. Limitations

The MATT test shows promising potential for assessing functional mobility in patients with PD. However, several limitations should be considered.

Firstly, the sample size is relatively small, which may constrain the generalizability of the findings to the broader PD population. Furthermore, limited diversity in the sample, particularly in terms of gender and disease stages, may impact the external validity of the study. A notable limitation in our study is the predominance of participants with mild-to-moderate PD. This focus restricts the applicability of the findings, as the MATT test might not adequately capture functional mobility in patients with advanced PD, who typically face more severe motor impairments and complex clinical challenges. Our participants were mostly classified as Hoehn and Yahr Stages 1 and 2, which corresponds to mild-to-moderate PD. This is consistent with the level of disease progression that is typically observed in ambulatory, community-dwelling individuals with PD. However, we are unable to comment on the suitability of this test for participants with more severe walking limitations. Future research should strive to include a wider range of disease severities to ensure the assessment tool’s utility across all stages of PD.

Another important consideration is that all assessments were conducted while participants were in the “ON” medication state. This requires careful control of medication effects to ensure that the results reflect functional mobility rather than fluctuations associated with medication status. Investigating whether the MATT test is sensitive to medication state transitions or improvements from physical therapy programs would provide valuable insights into its broader applicability. Additionally, the inclusion of a control group is a limitation of the study, as its presence would help establish or improve certain psychometric properties, such as discriminant validity. This would be especially valuable for comparing subjects in the “ON” state with healthy individuals.

Finally, the absence of long-term follow-up precludes evaluation of the test’s stability and reliability over time in PD patients. Addressing these limitations in future research will strengthen the evidence supporting the MATT test and enhance its applicability in diverse clinical and research settings.

## 6. Conclusions

The MATT test is a simple and efficient clinical timed test designed to evaluate specific functional mobility in individuals with PD. The MATT test and its segments showed good clinimetric properties and adequate validity. Clinimetric analysis reported excellent intra-rater, inter-rater, and test–retest reliability. The concurrent validity analysis showed a high-to-very-high correlation with the main tools historically used to evaluate functional mobility in PD patients.

In terms of clinical applicability, the MATT test required a short administration time and was well-understood by participants. Overall, these findings support the use of the MATT test as an effective assessment tool for mobility function in patients with mild-to-moderate PD. Additional studies are necessary to validate its use in patients at more severe stages of the disease.

## 7. Application Development

This section presents the development of a mobile application specifically designed for the implementation of the MATT. The application aims to facilitate test administration and enhance the user experience by providing an intuitive and accessible interface. Through this tool, healthcare professionals can administer evaluations more efficiently, while patients have easier access to the tests. The app is available for use on Android devices, and can be accessed via the following URL: https://drive.google.com/drive/folders/1P7mSa1ugopz3ImW7UkW7eZ6G4YQSVTID?usp=drive_link (accessed on 5 January 2025). A QR code is also provided for direct downloading: 
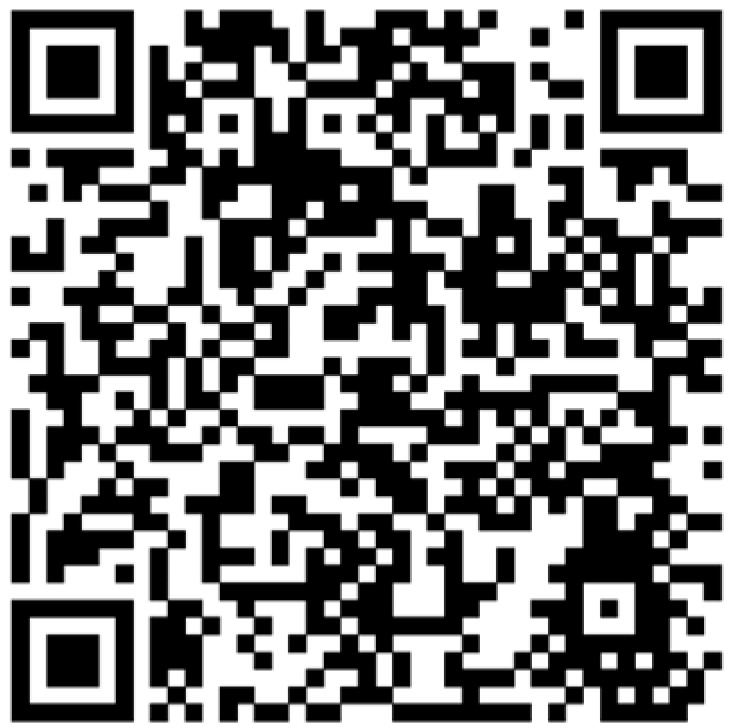


## Figures and Tables

**Table 1:** Participant characteristics.

Characteristics (Mean ± SD)	Participants (*n* = 57; 38 Men and 19 Women)
Age (years)	68.67 ± 8.49
BMI (kg/m^2^)	26.47 ± 3.52
Education (years)	10.84 ± 3.68
Disease duration (years)	6.16 ± 4.05
6-M retrospective falls (N)	2.51 ± 2.81
MMSE (0–30)	26.71 ± 2.46
H&Y (1–5)	2.21 ± 0.90
MDS-UPDRS-III (0–132)	32.95 ± 17.75

BMI = Body Mass Index; 6-M retrospective falls = 6-month retrospective falls; MMSE = Mini-Mental Status Examination Score; H&Y = Hoehn and Yahr scale; MDS-UPDRS III = modified Unified Parkinson Disease Rating Scale motor section.

**Table 2:** MATT score according to PD stage.

	MATT Score			
	Total Sample (*n* = 57)	Mild (*n* = 37)	Moderate (*n* = 15)	Severe (*n* = 5)
**Total time (s)**	55.11 ± 38.53	40.48 ± 11.68 ^†^	74.57 ± 53.45	122.32 ± 47.64 ^#^
**Segment 1 (s)**	21.41 ± 16.07	16.18 ± 5.00 ^†^	30.07 ± 26.62	39.56 ± 12.19 ^#^
**Segment 2 (s)**	22.23 ± 18.57	15.31 ± 4.65 ^†^	29.93 ± 19.92	59.28 ± 39.08 ^#^
**Segment 3 (s)**	11.46 ± 6.69	8.99 ± 2.55	14.57 ± 9.36	23.48 ± 5.99 ^#^

Note. ^†^ = significant difference between mild and moderate group; ^#^ = significant difference between mild and severe group. Significance: *p* < 0.05.

**Table 3:** Inter-segments correlation and correlation of each segment with the total time.

	Segment 1	Segment 2	Segment 3	Total Time
**Segment 1**	-	0.93 **	0.83 **	0.96 **
**Segment 2**		-	0.83 **	0.98 **
**Segment 3**			-	0.90 **
**Total time**				-

Note. ** = Significant difference at *p* < 0.01.

## Data Availability

Data can be obtained through the corresponding author upon reasonable request.

## Supplementary Materials

The following supporting information can be downloaded at: https://www.mdpi.com/article/10.3390/jcm14020361/s1. Supplemental Material S1. Characteristics of the participants; Supplemental Material S2. Intra-rater reliability data; Supplemental Material S3. Bland–Altman plots for the MATT scores recorded by the rater 1 on week 1 and 2. Solid central line represents mean between weeks (systematic bias); upper and lower dashed lines show mean ± 1.96 SD (random error); Supplemental Material S4. Inter-rater reliability data; Supplemental Material S5. Bland–Altman plots for inter-rater reliability. Y-axis is the difference between the MATT scores as recorded by the rater 1 and the rater 2 and X-axis is the mean of the MATT scores as recorded by the rater 1 and the rater 2. Solid central line represents mean between raters; upper and lower dashed lines show mean ± 1.96 SD (random error); Supplemental Material S6. Intra-session reliability; Supplemental Material S7. Bland–Altman plots for intra-session reliability. Y-axis is the difference between the MATT scores as recorded by the rater 1 on trial 1, 2 or 3 (T1, T2 or T3) and X-axis is the mean of the MATT scores as recorded by the rater 1 on trial 1, 2 or 3 (T1, T2 or T3). Solid line in the center is the mean change of score; upper and lower dashed lines show mean ± 1.96 SD (random error); Supplemental Material S8. Concurrent validity (correlation r/ρ) of MATT test with clinical test and laboratory parameters; Supplemental Material S9. Summary of clinimetric data: MATT test.
