# Supplementary material for: Motor Assessment Timed Test (MATT): A New Timed Test to Assess Functional Mobility in Parkinson’s Disease Patients
Source: J Clin Med. 2025 Jan 9;14(2):361. doi: 10.3390/jcm14020361 (PMC11765943; doi:10.3390/jcm14020361)
Supplement: Supplementary file 1 [file jcm-14-00361-s001.zip › Supplemental material S1.pdf]

Supplemental material S1. Characteristics of the participants.

Patient number	Sex	Age (years)	BMI	Education level (years)	Disease duration (years)	H&Y (1-5)	UPDRS-III (0 - 132)	Medication per day (mg)	LED (mg)
1	M	60	29.05	12	11	2	28	Levodopa/Benserazida 1000/250, Pramipexole 0.36	1036
2	M	72	27.10	5	5	2	20	Levodopa/Benserazide 800/200	800
3	M	74	21.36	5	11	3	50	Levodopa/Benserazide 1500/375	1500
4	F	69	34.60	6	5	1	7	Carbidopa/Levodopa 50/500, Pramipexole 0.18, Resagiline 1	618
5	M	70	29.69	5	12	2	10	Carbidopa/Levodopa 100/1250	1250
6	M	73	20.70	16	9	1	11	Carbidopa/Levodopa 100/400	400
7	M	74	25.10	10	6	1	12	Carbidopa/Levodopa 50/500, Pramipexole 0.18	518
8	F	71	25.56	10	2	2	18	Carbidopa/Levodopa 75/750, Ropirinole 1	770
9	F	70	29.30	12	15	3	24	Duodopa (13 ml morning + 3.5 ml continuous), Levodopa/Benserazide 100/25, Pramipexole 1.05	1585
10	M	83	22.77	6	4	4	69	Levodopa/Benserazide 600/150	600
11	F	73	28.30	12	6	2	23	Carbidopa/Levodopa 150/600, Ropirinole 1,5	630
12	M	71	31.24	14	3	1	10	Carbidopa/Levodopa 112.5/450, Pramipexole 1.57	607
13	M	44	26.08	10	8	2	16	Carbidopa/Levodopa 75/300, Pramipexole 0.25	325
14	M	84	23.11	14	3	2	27	Carbidopa/Levodopa 100/450	450
15	M	79	24.34	14	2	1	11	Levodopa/Benserazide 800/200, Carbidopa/Levodopa 50/200	1000
16	M	72	24.62	6	3	2	25	Levodopa/Benserazide 450/112,5	450
17	M	72	26.81	8	1	3	31	Carbidopa/Levodopa 75/750	750
18	M	73	22.77	12	9	3	39	Carbidopa/Levodopa 175/700	700
19	M	62	23.53	16	8	1	12	Carbidopa/Levodopa 75/300, Pramipexole 1.05, Resagiline 1	505
20	M	79	24.56	14	1	4	70	Levodopa/Benserazide 600/150	600
21	M	76	28.89	5	5	2	45	Carbidopa/Levodopa 75/300, Carbidopa/Levodopa 50/200	500
22	F	70	27.34	14	3	1	12	Levodopa/Benserazida 800/200, Carbidopa/Levodopa 50/200, Rotigotina 6	1180
23	M	61	32.39	10	3	2	17	Levodopa/Benserazida 300/75	300
24	F	58	30.49	16	6	3	31	Carbidopa/Levodopa 175/1750	1750
25	F	69	29.41	12	5	2	39	Levodopa/Benserazida 600/150	600
26	M	76	22.46	14	1	4	48	Carbidopa/Levodopa 75/750	750
27	M	72	26.84	6	3	2	53	Levodopa/Benserazide 600/150	600
28	F	71	23.45	10	2	1	10	Pramipexole 1.05, Resagiline 1	205
29	M	74	25.93	16	2	3	65	Carbidopa/Levodopa 75/750	750

Supplemental material S1. (continued)

Patient number	Sex	Age (years)	BMI	Education level (years)	Disease duration (years)	H&Y (1-5)	UPDRS-III (0 - 132)	Medication per day (mg)	LED (mg)
30	F	72	25.28	12	5	3	59	Carbidopa/Levodopa 25/250, Levodopa/Benserazide 300/75, Opicapone 50	575
31	M	64	22.79	6	10	1	10	Levodopa/Benserazida 450/112,5, Pramipexole 2,1	660
32	M	78	20.20	6	0.67	1	16	Levodopa/Benserazida 600/150	600
33	M	73	32.85	12	8	2	48	Levodopa/Benserazida 600/150, Pramipexole 1.5, Rotigotina 8, Carbidopa/Levodopa 25/100, Safinamide 50	1140
34	M	75	20.66	16	16	2	51	Levodopa/Carbidopa/Entacapone 400/100/800, Carbidopa/Levodopa 100/1000	1664
35	M	64	26.13	10	8	1	18	Levodopa/Benserazide 600/150	600
36	F	59	26.96	12	0.5	4	67	Carbidopa/Levodopa 112.5/1.125	1.125
37	F	60	34.02	12	5	1	14	Carbidopa/Levodopa 75/750, Carbidopa/Levodopa 50/200, Rotigotine 8, Rotigotine 3.	1.280
38	M	73	27.72	16	3	2	46	Levodopa/Benserazida 600/150	600
39	F	53	27.70	10	2	1	16	Resagilina 1, Carbidopa/Levodopa 137,5/550, Carbidopa/Levodopa 50/200	850
40	F	66	29.053	16	15	3	28	Carbidopa/Levodopa 125/500, Carbidopa/Levodopa 50/200, Pramipexole 210, Opicapone 50	935
41	M	66	27.10	10	11	3	63	Carbidopa/Levodopa 125/500,	500
42	M	44	21.36	10	6	2	58	Carbidopa/Levodopa 100/400, Carbidopa/Levodopa 50/200, Ropinirole 8, Safinamide 50, Opicapone 50	835
43	F	61	34.60	10	1	2	26	Resagiline 1, Carbidopa/Levodopa 75/750, Pramipexole 0.52	902
44	M	52	29.69	12	12	3	38	Carbidopa/Levodopa 62.5/625, Safinamide 50, Amantadine 100, Opicapone 50, apomorfine 10	900
45	M	68	20.70	10	10	3	33	Carbidopa/Levodopa 150/1500, Carbidopa/Levodopa 50/200, Ropinirol 8, Safinamide 100, Opicapona 50	1985
46	M	71	25.10	6	8	2	42	Carbidopa/Levodopa 175/700, Safinamide 50, Rotigotine 4	870
47	M	63	25.56	6	7	3	56	Carbidopa/Levodopa 112.5/450, Carbidopa/Levodopa 50/200, Safinamide 50, Opicapone 50, Pramipexole 1.05	830
48	M	69	29.30	16	6	2	31	Levodopa/Benserazida 800/200, Safinamide 50, Amantadine 200	1050
49	F	72	22.77	12	14	3	37	Resagiline 1.5, Carbidopa/Levodopa 37.5/150	300
50	F	70	28.30	6	11	3	30	Carbidopa/Levodopa 125/1250, Pramipexole 0.26, Resagiline 1	1376
51	M	72	31.24	6	3	2	32	Carbidopa/Levodopa 125/1250, Pramipexole 0.26	1276
52	M	79	26.08	16	2	2	33	Carbidopa/Levodopa 37.5/375	375

Supplemental material S1. (continued)

Patient number	Sex	Age (years)	BMI	Education level (years)	Disease duration (years)	H&Y (1-5)	UPDRS-III (0 - 132)	Medication per day (mg)	LED (mg)
53	M	73	23.11	10	4	2	32	Levodopa/Carbidopa/Entacapone 400/75/600, Rotigotine 8, Safinamide 50	888
54	M	54	24.34	12	1	2	32	Carbidopa/Levodopa 100/1000, Pramipexole 0.52	1052
55	F	74	24.62	16	3	2	48	Levodopa/Benserazida 400/100, Ropinirole 2	440
56	F	67	25.00	12	5	3	31	Levodopa/Carbidopa 400/50, Pramipexole 0.18	418
57	F	70	28.00	10	7	4	50	Levodopa/Carbidopa/Entacapone 800/200/800, Pramipexole 3.15	1379

Note. BMI = Body Mass Index; H&Y = Hoehn &Yahr scale; MDS-UPDRS (III) = modified Unified Parkinson Disease Rating Scale part III (motor examination); LED = Levodopa Equivalent Dose.
