# Supplementary material for: Motor Assessment Timed Test (MATT): A New Timed Test to Assess Functional Mobility in Parkinson’s Disease Patients
Source: J Clin Med. 2025 Jan 9;14(2):361. doi: 10.3390/jcm14020361 (PMC11765943; doi:10.3390/jcm14020361)
Supplement: Supplementary file 1 [file jcm-14-00361-s001.zip › Supplemental material S2.pdf]

Supplemental material S2. Intra-rater reliability data.

	Intra-rater reliability (Rater 1: week 1 and 2)				
	α Cronbach	ICC (95% CI)	CV %	SEM (%)	MDC_{95} (%)
**Total time (average)**	0.999	0.999**(0.999-0.999)	0.30	1.22 (2.21)	3.37 (6.12)
T1-T1	0.999	0.999**(0.999-0.999)	0.43	1.19 (2.06)	3.29 (5.71)
T2-T2	0.999	0.999**(0.999-0.999)	0.38	1.19 (2.20)	3.30 (6.10)
T3-T3	0.999	0.999**(0.999-0.999)	0.34	1.28 (2.29)	3.56 (6.36)
**Segment 1 (average)**	0.999	0.999**(0.999-0.999)	0.36	0.51 (2.36)	1.41 (2.55)
T1-T1	0.999	0.999**(0.999-0.999)	0.57	0.53 (2.38)	1.74 (6.60)
T2-T2	0.999	0.999**(0.999-0.999)	0.54	0.45 (2.17)	1.24 (6.02)
T3-T3	0.999	0.999**(0.999-0.999)	0.48	0.56 (2.61)	1.56 (7.23)
**Segment 2 (average)**	0.999	0.999**(0.999-0.999)	0.42	0.59 (2.64)	1.62 (7.31)
T1-T1	0.999	0.999**(0.999-0.999)	0.69	0.58 (2.50)	1.61 (6.93)
T2-T2	0.999	0.999**(0.999-0.999)	0.50	0.59 (2.66)	1.64 (7.37)
T3-T3	0.999	0.999**(0.999-0.999)	0.56	0.59 (2.79)	1.64 (7.74)
**Segment 3 (average)**	0.999	0.999**(0.998-0.999)	1.17	0.21 (1.86)	0.58 (5.15)
T1-T1	0.999	0.998**(0.996-0.999)	1.50	0.26 (2.21)	0.73 (6.13)
T2-T2	0.999	0.999**(0.998-0.999)	1.57	0.23 (2.04)	0.64 (5.65)
T3-T3	0.999	0.999**(0.998-0.999)	1.38	0.22 (2.07)	0.62 (5.73)
