# Supplementary material for: Motor Assessment Timed Test (MATT): A New Timed Test to Assess Functional Mobility in Parkinson’s Disease Patients
Source: J Clin Med. 2025 Jan 9;14(2):361. doi: 10.3390/jcm14020361 (PMC11765943; doi:10.3390/jcm14020361)

**Supplemental material S3.** Bland–Altman plots for the MATT scores recorded by the rater 1 on week 1 and 2. Solid central line represents mean between weeks (systematic bias); upper and lower dashed lines show mean ± 1.96 SD (random error).

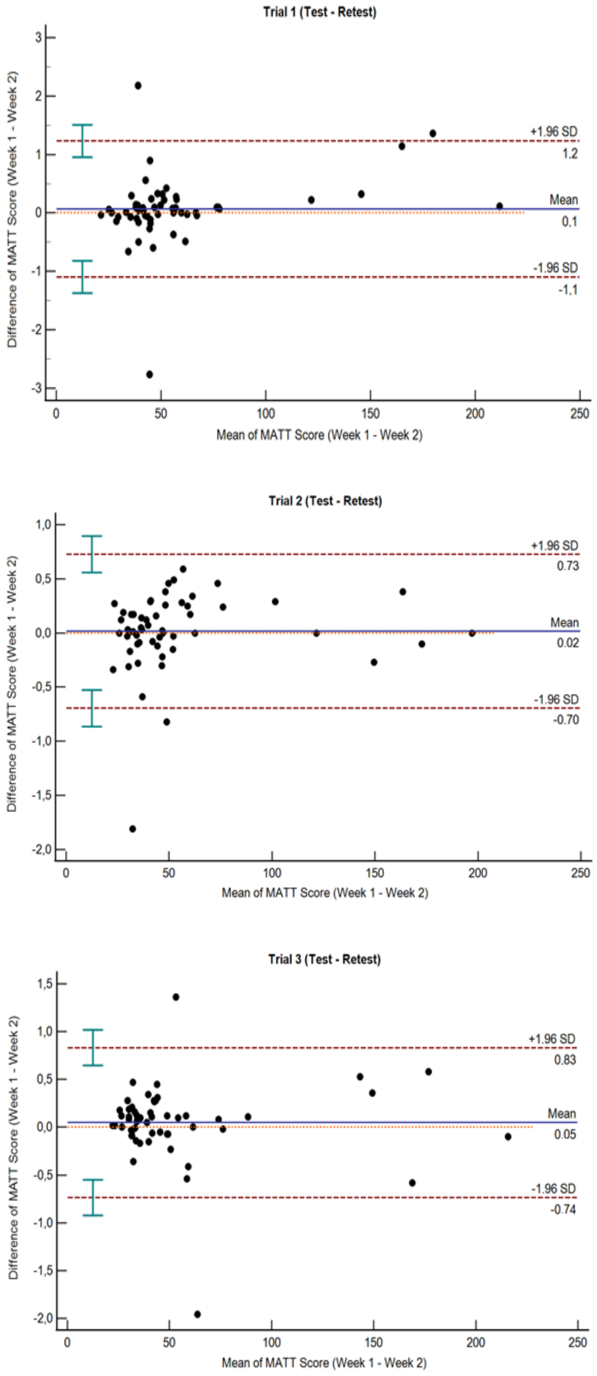

Supplement: Supplementary file 1 [file jcm-14-00361-s001.zip › Supplemental material S3.pdf]
