# Supplementary material for: Motor Assessment Timed Test (MATT): A New Timed Test to Assess Functional Mobility in Parkinson’s Disease Patients
Source: J Clin Med. 2025 Jan 9;14(2):361. doi: 10.3390/jcm14020361 (PMC11765943; doi:10.3390/jcm14020361)
Supplement: Supplementary file 1 [file jcm-14-00361-s001.zip › Supplemental material S4.pdf]

Supplemental material S4. Inter-rater reliability data.

	Inter-rater reliability (Rater 1 and 2)				
	**α Cronbach**	**ICC (95% CI)**	**CV %**	**SEM (%)**	**MDC_{95} (%)**
**Total time (average)**	0.998	0.992**(0.987-0.999)	0.91	1.22 (2.20)	3.37 (6.11)
T1-T1	0.999	0.999**(0.999-0.999)	0.31	1.19 (2.06)	3.29 (5.71)
T2-T2	0.999	0.999**(0.999-0.999)	0.19	1.19 (2.20)	3.30 (6.10)
T3-T3	0.999	0.999**(0.999-0.999)	0.25	1.28 (2.40)	3.56 (6.65)
**Segment 1 (average)**	0.999	0.999**(0.999-0.999)	0.35	0.51 (2.36)	1.41 (6.55)
T1-T1	0.996	0.979**(0.968-0.993)	1.18	0.53 (2.38)	1.47 (6.60)
T2-T2	0.999	0.999**(0.999-0.999)	0.47	0.45 (2.17)	1.24 (6.02)
T3-T3	0.999	0.999**(0.999-0.999)	0.49	0.56 (2.61)	1.56 (7.23)
**Segment 2 (average)**	0.998	0.995**(0.989-0.999)	0.72	0.59 (2.64)	1.62 (7.30)
T1-T1	0.999	0.999**(0.999-0.999)	0.63	0.58 (2.50)	1.61 (6.93)
T2-T2	0.999	0.999**(0.999-0.999)	0.57	0.59 (2.66)	1.64 (7.37)
T3-T3	0.999	0.999**(0.999-0.999)	0.56	0.59 (2.79)	1.64 (7.74)
**Segment 3 (average)**	0.997	0.985**(0.976-0.999)	1.11	0.21 (1.84)	0.59 (5.11)
T1-T1	0.997	0.976**(0.967-0.998)	1.42	0.19 (1.56)	0.52 (4.33)
T2-T2	0.995	0.965**(0.951-0.999)	1.65	0.23 (2.02)	0.64 (5.60)
T3-T3	0.995	0.977**(0.964-0.999)	1.45	0.22 (2.04)	0.61 (5.66)
