# Supplementary material for: Motor Assessment Timed Test (MATT): A New Timed Test to Assess Functional Mobility in Parkinson’s Disease Patients
Source: J Clin Med. 2025 Jan 9;14(2):361. doi: 10.3390/jcm14020361 (PMC11765943; doi:10.3390/jcm14020361)
Supplement: Supplementary file 1 [file jcm-14-00361-s001.zip › Supplemental material S6.pdf]

Supplemental material S6. Intra-session reliability.

	Rater 1					Rater 2				
	**α Cronbach**	**ICC (95% CI)**	**CV %**	**SEM (%)**	**MDC_{95} (%)**	**α Cronbach**	**ICC (95% CI)**	**CV %**	**SEM (%)**	**MDC_{95} (%)**
**Total time**										
T1-T2-T3	0.996	0.985**(0.972-0.992)	7.74	4.73 (8.59)	13.12 (23.82)	0.996	0.984**(0.971-0.991)	8.11	4.89 (8.88)	13.57 (24.61)
T1-T2	0.995	0.986**(0.957-0.994)	6.91	4.45 (7.97)	12.35 (22.10)	0.995	0.985**(0.953-0.993)	7.13	4.61 (8.23)	12.77 (22.82)
T1-T3	0.993	0.981**(0.944-0.992)	9.29	5.39 (9.71)	14.95 (26.91)	0.993	0.980**(0.941-0.991)	9.60	5.55 (9.98)	15.39 (27.68)
T2-T3	0.994	0.989**(0.981-0.993)	4.42	4.11 (7.63)	11.39 (21.12)	0.994	0.988**(0.979-0.993)	4.73	4.30 (7.98)	11.91 (22.12)
**Segment 1**										
T1-T2-T3	0.985	0.953**(0.928-0.971)	7.02	3.53 (16.45)	9.79 (45.59)	0.984	0.952**(0.925-0.970)	7.45	3.58 (16.77)	9.92 (46.48)
T1-T2	0.986	0.967**(0.930-0.983)	6.62	2.82 (13.15)	7.81 (36.44)	0.985	0.964**(0.922-0.982)	7.09	2.94 (13.818)	8.15 (38.30)
T1-T3	0.978	0.956**(0.926-0.974)	7.16	3.62 (16.53)	10.04 (45.82)	0.978	0.956**(0.926-0.974)	7.51	3.65 (16.69)	10.10 (46.26)
T2-T3	0.968	0.938**(0.896-0.963)	5.27	3.99 (18.95)	11.07 (52.54)	0.967	0.935**(0.891-0.962)	4.82	4.10 (19.60)	11.37 (54.33)
**Segment 2**										
T1-T2-T3	0.997	0.987**(0.975-0.993)	6.24	2.12 (9.57)	5.89 (26.52)	0.996	0.986**(0.972-0.992)	10.07	2.20 (9.91)	6.11 (27.47)
T1-T2	0.995	0.988**(0.977-0.993)	5.87	2.03 (8.95)	5.64 (24.81)	0.994	0.987**(0.975-0.993)	8.49	2.12 (9.29)	5.87 (25.75)
T1-T3	0.994	0.982**(0.932-0.993)	6.91	2.50 (11.24)	6.92 (31.16)	0.994	0.980**(0.923-0.992)	11.27	2.62 (11.80)	7.28 (32.71)
T2-T3	0.996	0.991**(0.981-0.996)	6.62	1.77 (8.18)	4.92 (22.69)	0.996	0.990**(0.979-0.995)	6.71	1.88 (8.64)	5.20 (23.96)
**Segment 3**										
T1-T2-T3	0.985	0.949**(0.914-0.970)	11.38	1.53 (13.45)	4.25 (37.29)	0.985	0.948**(0.912-0.970)	11.82	1.55 (13.42)	4.29 (37.19)
T1-T2	0.966	0.931**(0.883-0.960)	10.53	1.76 (15.02)	4.87 (41.62)	0.968	0.935**(0.888-0.962)	10.47	1.70 (14.34)	4.71 (39.75)
T1-T3	0.973	0.932**(0.825-0.968)	13.42	1.69 (14.83)	4.69 (41.12)	0.974	0.931**(0.817-0.968)	13.71	1.71 (14.86)	4.75 (41.20)
T2-T3	0.991	0.978**(0.957-0.988)	6.54	1.06 (9.60)	2.95 (26.61)	0.989	0.974**(0.948-0.986)	7.60	1.15 (10.28)	3.19 (28.50)

* = Significant difference at $p < 0.05$; ** = Significant difference at $p < 0.01$; α Cronbach = Cronbach's alpha; ICC = Intraclass Correlation Coefficient, 95% CI = 95% Confidence Interval; CV % = percentage of Coefficient of Variation; SEM = Standard Error of Measurement; MDC_{95} = Minimal Detectable Change; (%) = MDC expressed as a percent of measurement mean; T1= trial 1; T2= trial 2; T3= trial 3.
