# Supplementary material for: Motor Assessment Timed Test (MATT): A New Timed Test to Assess Functional Mobility in Parkinson’s Disease Patients
Source: J Clin Med. 2025 Jan 9;14(2):361. doi: 10.3390/jcm14020361 (PMC11765943; doi:10.3390/jcm14020361)

Supplemental material S7. Bland–Altman plots for intra-session reliability. Y-axis is the difference between the MATT scores as recorded by the rater 1 on trial 1, 2 or 3 (T1, T2 or T3) and X-axis is the mean of the MATT scores as recorded by the rater 1 on trial 1, 2 or 3 (T1, T2 or T3). Solid line in the center is the mean change of score; upper and lower dashed lines show mean ± 1.96 SD (random error).

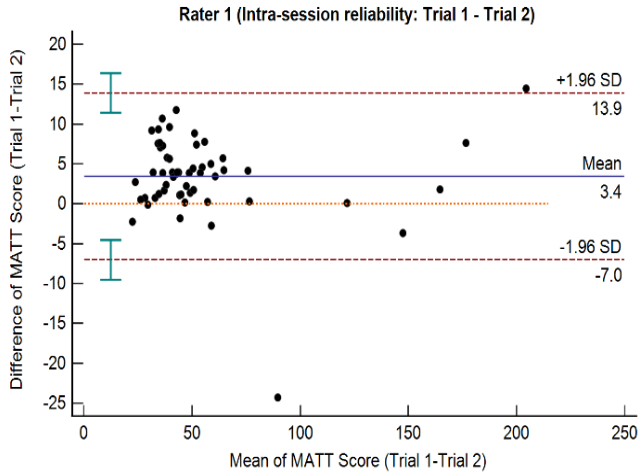

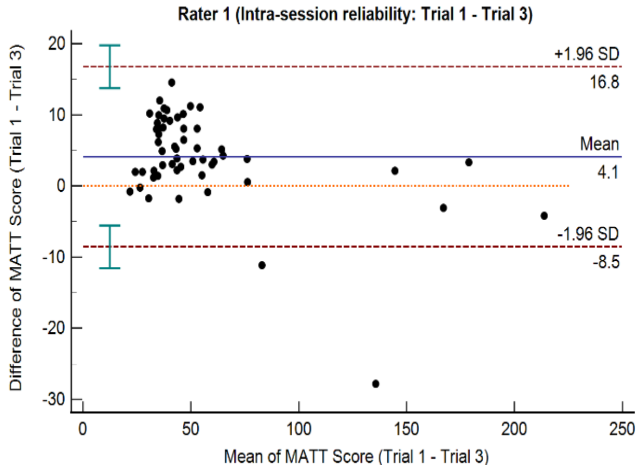

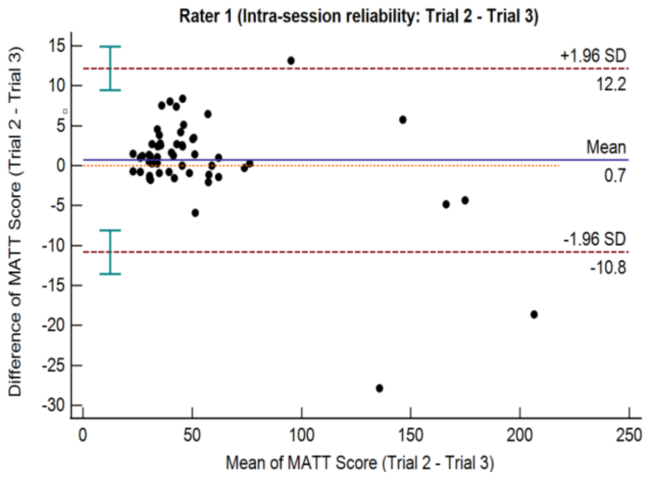

Supplement: Supplementary file 1 [file jcm-14-00361-s001.zip › Supplemental material S7.pdf]
