# Supplementary material for: Motor Assessment Timed Test (MATT): A New Timed Test to Assess Functional Mobility in Parkinson’s Disease Patients
Source: J Clin Med. 2025 Jan 9;14(2):361. doi: 10.3390/jcm14020361 (PMC11765943; doi:10.3390/jcm14020361)
Supplement: Supplementary file 1 [file jcm-14-00361-s001.zip › Supplemental material S8.pdf]

Supplemental material S8. Concurrent validity (correlation *r*/ϱ) of MATT test with clinical test and laboratory parameters.

Test Name	Segment 1			Segment 2			Segment 3			MATT Total time		
	r/ϱ	95% CI	*p*	*r*/ϱ	95% CI	*p*	*r*/ϱ	95% CI	*p*	*r*/ϱ	95% CI	*p*
H&Y (1-5)	0.64**	0.46 – 0.77	< 0.001	0.68**	0.51 – 0.80	< 0.001	0.54**	0.32 –0.72	< 0.001	0.66**	0.49 – 0.79	< 0.001
MDS-UPDRS (III) (0-132)	0.57**	0.32 – 0.74	< 0.001	0.62**	0.38 – 0.79	< 0.001	0.53**	0.29 –0.72	< 0.001	0.60**	0.37 – 0.77	< 0.001
Short FES-I (7-28)	0.51**	0.26 – 0.69	< 0.001	0.48**	0.20 – 0.68	< 0.001	0.41**	0.12 –0.63	0.002	0.47**	0.18 – 0.67	< 0.001
6-M retrospective falls	0.63**	0.41 – 0.79	< 0.001	0.65**	0.46 – 0.80	< 0.001	0.63**	0.41 –0.78	< 0.001	0.65**	0.44 – 0.80	< 0.001
FOG-Q (0-132)	0.52**	0.34 – 0.64	< 0.001	0.52**	0.39 – 0.68	< 0.001	0.41**	0.35 –0.70	< 0.001	0.50**	0.39 – 0.70	< 0.001
MMSE (0-30)	-0.50**	-0.68 – -0.29	< 0.001	-0.58**	-0.73 – -0.38	< 0.001	-0.52**	-0.68 – -0.30	< 0.001	-0.57**	-0.73 – -0.36	< 0.001
ABC scale (0-100)	-0.71**	-0.81 – -0.55	< 0.001	-0.69**	-0.82 – -0.51	< 0.001	-0.63**	-0.78 – -0.42	< 0.001	-0.70**	-0.81 – -0.51	< 0.001
TMT "A"(s)	0.42**	0.16 – 0.63	0.001	0.52**	0.25 – 0.71	< 0.001	0.53**	0.28 –0.73	< 0.001	0.51**	0.24 – 0.70	< 0.001
TMT "B" (s)	0.50**	0.29 – 0.66	< 0.001	0.57**	0.35 – 0.74	< 0.001	0.58**	0.38 –0.73	< 0.001	0.57**	0.35 – 0.72	< 0.001
TMT "B-A" (s)	0.48**	0.27 – 0.64	< 0.001	0.52**	0.30 – 0.70	< 0.001	0.55**	0.33 –0.70	< 0.001	0.53**	0.32 – 0.68	< 0.001
Tinetti "BS" (0-16)	-0.65**	-0.76 – -0.46	< 0.001	- 0.68**	-0.79 – -0.50	< 0.001	-0.55**	-0.73 – -0.31	< 0.001	-0.67**	-0.78 – -0.49	< 0.001
Tinetti "GS" (0-12)	-0.64**	-0.79 – -0.43	< 0.001	- 0.70**	-0.80 – -0.54	< 0.001	-0.51**	-0.69 – -0.25	< 0.001	-0.67**	-0.80 – -0.48	< 0.001
Tinetti "TS" (0-28)	-0.70**	-0.80 – -0.53	< 0.001	- 0.74**	-0.83 – -0.59	< 0.001	-0.57**	-0.74 – -0.32	< 0.001	-0.72**	-0.82 – -0.55	< 0.001
BBS (0-56)	-0.79**	-0.87 – -0.65	< 0.001	- 0.84**	-0.91 – -0.72	< 0.001	-0.72**	-0.86 – -0.52	< 0.001	-0.83**	-0.90 – -0.70	< 0.001
FRT (cm)	-0.42**	-0.53 – -0.16	0.001	- 0.40**	-0.54 – 0.01	0.002	-0.28*	-0.46 – 0.04	0.042	-0.38**	-0.55 – 0.07	0.004
10-MWT (m/s)	-0.88**	-0.79 – -0.93	< 0.001	- 0.83**	-0.70 – -0.90	< 0.001	-0.73**	-0.54 – -0.85	< 0.001	-0.85**	-0.73 – -0.92	< 0.001
TUG (s)	0.89**	0.79 – 0.95	< 0.001	0.83**	0.68 – 0.91	< 0.001	0.78**	0.60 – 0.88	< 0.001	0.86**	0.74 – 0.94	< 0.001
Cognitive TUG (s)	0.91**	0.82 – 0.96	< 0.001	0.89**	0.78 – 0.95	< 0.001	0.84**	0.71 – 0.90	< 0.001	0.91**	0.83 – 0.96	< 0.001
TE COP (OE) (mm)	0.37**	0.14 – 0.58	0.005	0.33*	0.07 – 0.56	0.015	0.33*	0.08 – 0.55	0.014	0.36**	0.11 – 0.58	0.006
MS COP (OE) (mm/s)	0.39**	0.15 – 0.58	0.004	0.34*	0.08 – 0.56	0.011	0.34*	0.09 – 0.55	0.012	0.38**	0.12 – 0.58	0.005
TE COP (CE) (mm)	0.45**	0.21 - 0.65	0.001	0.41**	0.14 – 0.62	0.002	0.36**	0.10 – 0.60	0.007	0.43**	0.17 – 0.65	0.001
MS COP (CE) (mm/s)	0.46**	0.22 - 0.65	< 0.001	0.41**	0.16 – 0.62	0.002	0.37**	0.12 – 0.60	0.006	0.44**	0.19 – 0.65	0.001

* = Significant difference at p < 0.05; ** = Significant difference at p < 0.01; ϱ = Spearman's rank correlation coefficient; H&Y = Hoehn &Yahr scale; MDS-UPDRS (III) = modified Unified Parkinson Disease Rating Scale part III (motor examination); short FES-I = short version of Falls Efficacy Scale-International; 6-M retrospective falls= subjective recall of number of falls in last 6 months; FOG-Q = Freezing of Gait Questionnaire; MMSE = Mini-Mental Scale Examination; ABC scale = Activities-specific Balance Confidence Scale; TMT "A" = Trail Making Test part A; TMT "B" = Trail Making Test part B; TMT "B-A" = Trail Making Test part B minus part A; Tinetti "BS" = Tinetti Balance Section; Tinetti "GS" = Tinetti Gait Section; Tinetti "TS" = Tinetti Balance Total Score; BBS= Berg Balance Scale;

FRT = Functional Reach Test; 10-MWT = 10 Meter Walking Test; TUG = Time Up and Go test; Cognitive TUG = time to complete the Time Up and Go test with an added simple cognitive task; TE COP (OE) = Center of pressure total trajectory length (Open Eyes); Mean Speed COP (OE) = Mean Speed of Center of Pressure (Open Eyes); TE COP (CE) = Center of pressure total trajectory length (Closed Eyes); MS COP (CE) = Mean Speed of Center of Pressure (Closed Eyes).
