# Supplementary material for: Motor Assessment Timed Test (MATT): A New Timed Test to Assess Functional Mobility in Parkinson’s Disease Patients
Source: J Clin Med. 2025 Jan 9;14(2):361. doi: 10.3390/jcm14020361 (PMC11765943; doi:10.3390/jcm14020361)
Supplement: Supplementary file 1 [file jcm-14-00361-s001.zip › Supplemental material S9.pdf]

**Supplemental material S9.** Summary of clinimetric data: MATT test.

| | MATT test | | | |
|---|---|---|---|---|
| **Clinimetric data** | **Segment 1** | **Segment 2** | **Segment 3** | **Total time** |
| Content Validity | ++++ | ++++ | +++ | ++++ |
| Criterion Validity | ++++ | ++++ | +++ | ++++ |
| Construct Validity | ++++ | ++++ | +++ | ++++ |
| Internal Consistency | ++++ | ++++ | ++++ | ++++ |
| Intra-rater reliability | ++++ | ++++ | ++++ | ++++ |
| Inter-rater reliability | ++++ | ++++ | ++++ | ++++ |
| Intra-session reliability | ++++ | ++++ | ++++ | ++++ |
| Responsiveness to change | N.E. | N.E. | N.E. | N.E. |
| $MDC_{95}$ | YES | YES | YES | YES |
| MCID | N.E. | N.E. | N.E. | N.E. |
| Floor effect | YES | NO | NO | NO |
| Ceiling effect | NO | NO | NO | NO |
| Learning effect | YES† | YES†# | YES# | YES†# |
| Skewness | YES | NO | NO | NO |
| Feasibility/Applicability (spectrum) | NO | NO | NO | NO |
| Feasibility/Applicability (length/completion) | +++ | +++ | +++ | ++ |
| Participants' comprehension | N.E. | N.E. | N.E. | +++ |
| Level of difficulty perceived | N.E. | N.E. | N.E. | +++ |

C-D = Convergent-Discriminant; $MDC_{95}$ = Minimal Detectable Change; MCID = Minimal Clinically Important Difference; N.E. = not evaluated; N.A. = not applicable; (-) = poor; (+) = weak; (++) = moderate; (+++) = good; (++++) = very good. † = significant difference between T1-T2; # = significant difference between T1-T3.
